# The Vena Azygos in Cloven Footed Animals

**Published:** 1889-07

**Authors:** R. W. Burke

**Affiliations:** Army Veterinary Department, Jubblepore, India


### Art. XIII.—THE VENA AZYGOS IN CLOVEN FOOTED ANIMALS.

By R. W. Burke, M.R.C.V.S.,
*Army Veterinary Department, Jubblepore, India.*

The relation of the large vena azygos with the other vessels differs in ruminants and swine from that found in the horse. In the latter, it most frequently enters the anterior vena cava and sometimes the right auricle directly, close to it. In the former, *it enters the right auricle below the posterior vena cava, occupying the place of the coronary vein.* It varies in its position also with reference to its relations with the right and left auricles in the different species. In the horse, it is seen to enter directly in relation with the right auricle; whereas, in the case of the artiodactyla, in descending from the spine, *it curves round in relation first with the left auricle, and receives the mouth of the coronary vein before it opens into the right auricle below the posterior vena cava.* In the horse, the vena azygos runs along the right side, and *in ruminants along the left side of the spine.*

The great coronary vein in the horse opens directly into the right auricle; in the artiodactyla it (or, rather, the smaller cardiac veins) *join the large vena azygos before that vessel enters the right auricle.*

The large vena azygos in larger ruminants as the cow and buffalo, is partly overlapped in its course by the posterior aorta; *in the smaller ruminants, as the goat, sheep and deer, it is visible throughout its whole course along the left side of the spine.*

In the horse, the large vena azygos runs along with the thoracic duct on the right side of the posterior aorta and spine. In cloven footed animals, it passes forward along the left side, running parallel with the posterior aorta and receiving the intercostal veins, till it reaches the spine opposite the base of the heart, where *it descends forming an angle in relation with the left auricle, before it receives the mouth of the coronary vein and enters the right auricle.*

The small vena azygos gives off one or more smaller anastomosing branches which open into the large vena azygos before it reaches the base of the heart. In the horse, the large vena azygos being situated always on the right side, there are no such anastomosing branches above described. Hence some anatomists have described the large vena azygos to be a prolongation of the smaller one.

After placing the animal on its back and opening the chest, in the case of cloven footed animals, turn the left lung towards the mediastinum and expose the vena azygos which will be seen running along the left side of the spine. On turning over the right lung to the mediastinum, no such vessel will be seen. In the horse, however, as is well known, the large vena azygos invariably takes the opposite side. In cloven footed animals the *venæ azygos, large and small, both occupy the left side of the spine, and frequently also anastomose by means of one or more smaller vessels.* Hence it is anatomists have, most probably, mistaken the large or great vena azygos to be a prolongation of the smaller one. In the horse, as we are already aware, the large vena azygos is found on the right side, and the small on the left. *There is no vena azygos on the right side in ruminants.*

It would appear that anatomists having seen the large vena azygos in ruminants on the left side of the spine, have taken it to be a prolongation of the small vena azygos, as is sometimes the case in horses. However this may be, we would submit the above notes for consideration.

